# Predictors of loss to follow up among patients with type 2 diabetes mellitus attending a private not for profit urban diabetes clinic in Uganda – a descriptive retrospective study

**DOI:** 10.1186/s12913-019-4415-4

**Published:** 2019-08-23

**Authors:** Salome Tino, Clara Wekesa, Onesmus Kamacooko, Anthony Makhoba, Raymond Mwebaze, Samuel Bengo, Rose Nabwato, Aisha Kigongo, Edward Ddumba, Billy N. Mayanja, Pontiano Kaleebu, Rob Newton, Moffat Nyerinda

**Affiliations:** 1 MRC/UVRI and LSHTM Uganda Research Unit, P.O. Box 49, Entebbe, Uganda; 2 St. Francis Hospital Nsambya, P. O. Box, 7146 Kampala, Uganda; 3 London School of Hygiene and Tropical Medicine, Keppel Street, London, WC1E 7HT United Kingdom; 4 Department of Health Sciences, University of York, Heslington, York, YO10 5DD United Kingdom

**Keywords:** Type 2 diabetes mellitus, Lost to follow up, Diabetes clinic, Uganda, Predictors

## Abstract

**Background:**

Although the prevalence of type 2 diabetes mellitus is increasing in Uganda, data on loss to follow up (LTFU) of patients in care is scanty. We aimed to estimate proportions of patients LTFU and document associated factors among patients attending a private not for profit urban diabetes clinic in Uganda.

**Methods:**

We conducted a descriptive retrospective study between March and May 2017. We reviewed 1818 out-patient medical records of adults diagnosed with type 2 diabetes mellitus registered between July 2003 and September 2016 at St. Francis Hospital - Nsambya Diabetes clinic in Uganda. Data was extracted on: patients’ registration dates, demographics, socioeconomic status, smoking, glycaemic control, type of treatment, diabetes mellitus complications and last follow-up clinic visit. LTFU was defined as missing collecting medication for six months or more from the date of last clinic visit, excluding situations of death or referral to another clinic. We used Kaplan-Meier technique to estimate time to defaulting medical care after initial registration, log-rank test to test the significance of observed differences between groups. Cox proportional hazards regression model was used to determine predictors of patients’ LTFU rates in hazard ratios (HRs).

**Results:**

Between July 2003 and September 2016, one thousand eight hundred eighteen patients with type 2 diabetes mellitus were followed for 4847.1 person-years. Majority of patients were female 1066/1818 (59%) and 1317/1818 (72%) had poor glycaemic control. Over the 13 years, 1690/1818 (93%) patients were LTFU, giving a LTFU rate of 34.9 patients per 100 person-years (95%CI: 33.2–36.6). LTFU was significantly higher among males, younger patients (< 45 years), smokers, patients on dual therapy, lower socioeconomic status, and those with diabetes complications like neuropathy and nephropathy.

**Conclusion:**

We found high proportions of patients LTFU in this diabetes clinic which warrants intervention studies targeting the identified risk factors and strengthening follow up of patients.

## Background

Globally, 422 million adults aged over 18 years were living with diabetes mellitus in 2014 and this is expected to rise to 552 million by 2030 [1]. Type 2 diabetes mellitus is becoming epidemic in nearly every population and it’s proposed that without effective prevention and control programmes, the prevalence will continue to increase globally most likely as a result of rising overweight and obesity rates, lifestyle, dietary changes, and an ageing population [2]. This condition is not only affecting developed countries but it’s rising rapidly in low and middle income countries [3, 4]. However, the health systems in low and middle income countries have mainly targeted healthcare provision for acute episodic conditions and not medical care for chronic conditions. In Uganda the prevalence of diabetes mellitus is estimated at 10.1% while impaired glucose tolerance is estimated at 13.8% [5].

Several studies conducted in developed countries have shown that continuous care and greater compliance to clinic appointments is associated with good glycaemic control, decreased probability of diabetes-related complications and mortality because patients who stop treatment miss opportunities for detecting complications and treatment adjustments [6–11]. However, in other developed countries the rates of patients dropping out from diabetes care are high and varied: 5.4 and 18% in Malaysia [12], 5.5% in German [13], 12% in Nashville-Tennessee [14] and 46% in Canada [15]. Quantitative studies done in the United Kingdom and United States showed that overweight or obesity, hypertension, presence of neuropathy, poor glycaemic control, older age, low income, smoking, rural residence and male sex were associated with clinic follow-up non-attendance [14, 16–21]. Another study revealed that men, not being on insulin or antiplatelet agents, having higher HbA1c, higher Low Density Lipoprotein Cholesterol (LDL-C) and having complications of diabetes mellitus were associated with follow-up non-attendance. Older age and higher LDL-C were also associated with higher mortality. Reports from Africa on LTFU are limited, but one study conducted in Kenya reported a dropout rate of 31% among registered patients with diabetes mellitus in a primary health care program [22]. In a recent systematic review of factors affecting follow-up non-attendance, 83 factors were classified into three categories. These included patient factors (e.g. mental state, alcohol and tobacco use, etc.); disease and medication factors (e.g. poor disease control); and health care provider factors (e.g. scheduling, health provider characteristics, doctor-patient relationships [23]. In Uganda, no study has estimated the LTFU among patients with diabetes mellitus and we theorize that rates of LTFU are high and its predictors are varied. Therefore, this descriptive retrospective study of clinic medical records through chart reviews was undertaken to estimate the LTFU rates and document the associated factors since this is an important determinant of poor glycaemic control long-term outcomes. The findings from this study will inform policy makers and program managers to address the challenges of patient LTFU as well as provide a base line report for interventions aimed at reducing LTFU in the management of diabetes mellitus.

## Methods

### Study design, setting and population

This was a descriptive retrospective study of 1818 selected medical records of adult patients with diabetes mellitus who were registered in the diabetes clinic at St. Francis Hospital Nsambya in Kampala-Uganda, which is a private not for profit facility [24]. The hospital conducts a diabetes mellitus clinic that operates once a week serving an average of 60 patients on each clinic day. In the diabetes clinic, all patients receive diabetes mellitus health education offered by trained diabetes nurses and fasting blood glucose measurements prior to being reviewed by the doctors [25]. The study population comprised of patients with type two diabetes mellitus who were registered at the clinic between July 2003 and September 2016. The charts were reviewed by a medical doctor, clinical officer and registered nurses for sociodemographic characteristics, date of registration, date of last clinic visit and clinical outcomes using a data collection guide. Patients whose paper clinic records files could not be located, those with missing information, transferred out or recorded as dead, type 1 diabetes mellitus, gestational diabetes were excluded.

### Definition of variables

LTFU: Patients were considered LTFU if they had missed collecting medication for six or more consecutive months from the date they last visited the clinic and retention in medical care considered as continuous follow-up visits to the same health-care service to seek treatment for the same episode of illness [26]. Transfer to other facilities or death of a patient were not regarded as LTFU.

Different treatment options were defined as follows: Life style alone was defined as non-pharmacological treatment involving changes in diet and increased exercise; monotherapy as use of metformin alone or any other single oral hypoglycemic; dual therapy as a combination of metformin plus another oral hypoglycemic or metformin plus insulin; triple therapy as a combination of metformin plus two other oral hypoglycemic agents or metformin plus another oral hypoglycemic and insulin; and combination injection therapy was defined as use of different insulin regimens.

Hypertension: This was defined as systolic blood pressure ≥ 140 mmHg and/or diastolic pressure of ≥90 mmHg.

Optimal glycaemic control: This was defined as pre-prandial glucose 4.4–7.2 mmol/L measured as an average of the last 3 readings on 3 separate clinic visits among patients with diabetes mellitus under care. Fasting glucose reading of > 7.2 mmol/L was considered as uncontrolled glycaemia.

Socioeconomic status (SES) was defined based on house hold assets. Upper SES defined as living in a house with electricity or solar power supply, piped water, flushing toilet and kitchen inside; Middle SES as living in a house with at least one but not all the utilities above; Lower SES as living in a house without the above and not mud or wattle and not grass hatched; Poor SES as living in mud and wattle grass hatched house and none of the amenities above.

For residence we used the host hospital's definations; urban was defined as living within 1 km (km) of a town council; Peri-urban as living within 1 km of a shop selling soft drinks and Rural as living more than 1 km of a shop selling soft drinks. 

### Data source and collection

The data for this research was secondary data collected routinely in the hospital for patients’ clinical monitoring and evaluation purposes. Data was collected and entered into a Microsoft Excel 2010 database (Microsoft Corp., Redmond, WA), checked for consistencies and completeness, and then exported to STATA 13.1 (Stata Corp, College Station, TX, USA) for further management and analysis. Data collected for this review included demographics, date of first registration, date of last clinic visit, past medical history, glycaemic levels, medication use, complications of diabetes and biophysical measurements. The primary outcome variable was LTFU from the diabetes mellitus care after initiation of treatment, confirmed by reviewing medical records at the hospital.

### Statistical analysis

The patients’ characteristics were described in terms of mean, median or percentage as appropriate. The Kaplan-Meier technique was used to estimate time to defaulting medical care after initial registration into the diabetes clinic, while the log-rank test was used to test the significance of observed differences between groups. The Cox proportional hazards regression model was used to determine predictors of patients’ retention and defaulting rates expressed as estimated hazard ratios (HRs) with their corresponding 95% confidence intervals (CIs). At the unadjusted modelling level, variables which gave a Log- Likelihood ratio tests (LRTs) *p*-value less than or equal to 0.1 were considered for the adjusted model. At the multivariable analysis, variables that were earlier dropped, were again added onto the model one by one to look at the effect until the final model was obtained. Variables whose p-value was less than 0.05 level were considered to be independent risk factors of LTFU in this study. We considered age and sex as priori confounders.

## Results

**Fig. 1:**
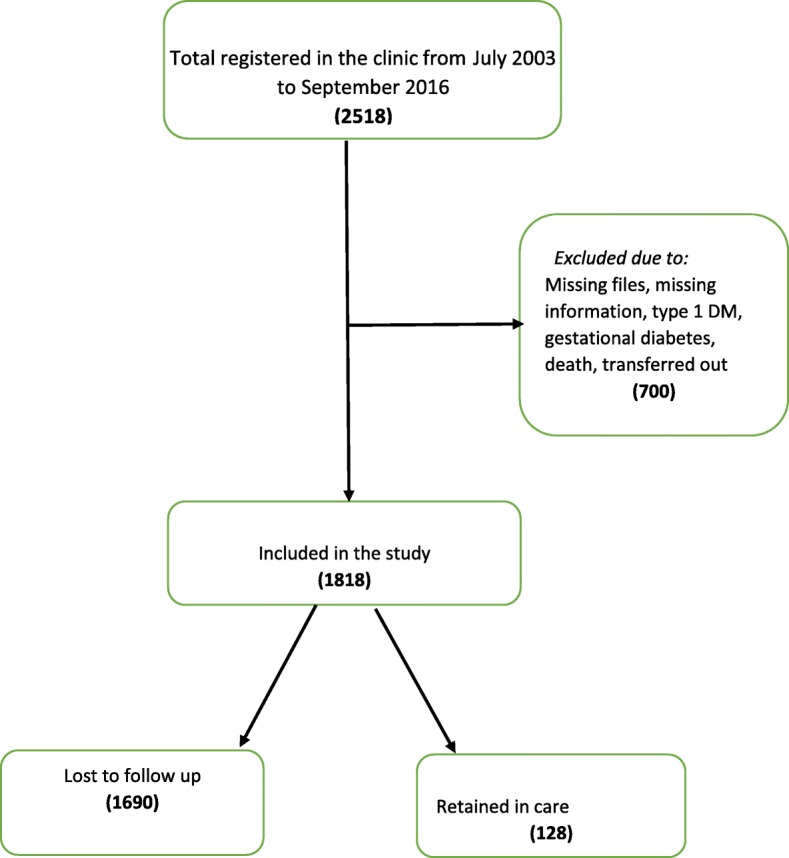
Study organogram

Between July 2003 and September 2016, a total of 2518 diabetes mellitus patients were registered in the diabetes clinic of St. Francis Hospital Nsambya in Kampala, Uganda. We excluded 700 records due to missing files, files with missing information, type 1 diabetes mellitus, gestational diabetes mellitus, deaths and transfers out (Fig. 1). The 1818 patients included in the study were followed for a total of 4847.1 person-years, with the longest individual follow up being 14.2 years. The majority of the patients were females 59% (*n* = 1066), 75% were aged more than 45 years old (*n* = 1364), 89% resided in peri-urban and urban areas (*n* = 1611), 76% belonged to middle and upper social class (*n* = 1328), about 10% were smokers (*n* = 190), 26% were alcohol consumers (*n* = 466), 18% had retinopathy (*n* = 330), 66% had uncontrolled hypertention (*n* = 1199) and 72% (*n* = 1317) had poor glycaemic control (Table 1).

**Table 1:** Baseline characteristics of patients retained and LTFU in a retrospective chart review study of patients with type 2 diabetes mellitus

Variable Name	Category	Retained N (col %) 128 (7%)	LTFU N (col %) 1690 (93%)	Chi-Square *P*-value
Socio-demographic characteristics				
Sex				**0.016**
	Female	88 (69)	978 (58)	
	Male	40 (31)	712 (42)	
Median age (IQR) (years)	Age	60 (50–68)	53 (44–64)	**< 0.001**
Age group (years)				**0.001**
	19–44	15 (12)	439 (26)	
	45–64	68 (53)	831 (49)	
	Above 64	45 (35)	420 (25)	
Residence				0.966
	Rural	14 (11)	193 (12)	
	Peri-urban	33 (26)	422 (24)	
	Urban	81 (63)	1075 (64)	
Education level				0.23
	<=Primary Education	50 (39)	753 (45)	
	Secondary	53 (41)	574 (34)	
	Post-Secondary	25 (20)	363 (21)	
Socioeconomic status				0.107
	Lower	22 (17)	408 (24)	
	Middle	58 (45)	771 (46)	
	Upper	48 (38)	511 (30)	
Life style characteristics				
Smoking				0.19
	No	119 (93)	1509 (89)	
	Yes	9 (7)	181 (11)	
Alcohol				0.646
	No	93 (73)	431 (26)	
	Yes	35 (27)	1259 (74)	
DM treatment characteristics				
Life style alone				0.176
	No	127 (99)	1637 (97)	
	Yes	1 (1)	53 (3)	
Lifestyle and monotherapy				**0.03**
	No	106 (83)	1254 (74)	
	Yes	22 (17)	436 (26)	
Lifestyle and dual therapy				**0.006**
	No	40 (31)	740 (44)	
	Yes	88 (69)	950 (56)	
Lifestyle and triple therapy				0.359
	No	115 (90)	1557 (92)	
	Yes	13 (10)	133 (8)	
Lifestyle and combination injection therapy				**0.037**
	No	122 (95)	1514 (90)	
	Yes	6 (5)	176 (10)	
DM complications and comorbidities				
Neuropathy				**< 0.001**
	No	54 (42)	1141 (68	
	Yes	74 (58)	549 (32))	
Nephropathy				**0.005**
	No	114 (89)	1605 (95)	
	Yes	14 (11)	85 (5)	
Retinopathy				**0.01**
	No	94 (73)	1394 (82)	
	Yes	34 (27)	296 (18)	
Macroangiopathy				**0.003**
	No	120 (94)	1654 (98)	
	Yes	8 (6)	36 (2)	
Diabetic foot				**0.038**
	No	116 (91)	1604 (95)	
	Yes	12 (9)	86 (5)	
Hypoglycaemia				0.095
	No	122 (95)	1651 (98)	
	Yes	6 (5)	39 (2)	
Hypertension				**0.001**
	No	26 (20)	593 (35)	
	Yes	102 (80)	1097 (65)	
Erectile dysfunction^a^				**< 0.001**
	No	23 (57)	576 (81)	
	Yes	17 (43)	136 (19)	
Fasting plasma glucose (Mmol/l)				0.955
	Controlled glycaemia	35 (27)	466 (28)	
	Hyperglycaemia	93 (73)	1224 (72)	

*DM* - Diabetes mellitus, ^a^Erectile dysfunction - (Not Applicable for 1066 females). Boldface means significant at *P*-value < 0.05 for Tables 1 & 2

Over the 13 years follow up, 93% (*n* = 1690) of the patients were lost to follow up, with 52% (*n* = 945) of the LTFU occurring within the first year after registration in the diabetes clinic. The median time to LTFU was 11 months; Interquartile range 5.9 to 45.0 months. The estimated LTFU rate was 34.9 per 100 person-years (95% CI: 33.2 – 36.6). In the multivariate analysis, male patients were more likely to be LTFU than females (adjusted Hazard Ratio, aHR 1.17 [95% CI: 1.06–1.30]). Patients aged 45–64 years (aHR 0.83; 95% CI: 0.74–0.94) and those above 64 years (aHR 0.78; 95% CI: 0.67–0.90) were more likely to be retained in care than the younger age group of 44 years and below. Retention in diabetes mellitus care was also associated with middle socioeconomic status (aHR 0.83; 95% CI: 0.74–0.94) and upper social economic status (aHR 0.83; 95% CI: 0.73–0.95) compared to  lower socioeconomic status, being a non-smoker (aHR 0.82; 95% CI: 0.70–0.96) and being on dual therapy (aHR 0.78; 95% CI: 0.70–0.86). Loss to follow up from care was associated with diabetes mellitus complications such as neuropathy (aHR 1.38; 95% CI: 1.24–1.54), nephropathy (aHR 1.76; 95% CI: 1.41–2.20), being on triple therapy (aHR of 1.30; 95% CI: 1.03–1.63). Patients on lifestyle control alone and combination injection therapy were few and were not associated with loss to follow up. Residence, education level, alcohol intake, lifestyle therapy alone, combination injection therapy, complications of diabetes mellitus such as hypertension, retinopathy, macroangiopathy (coronary artery disease, cerebral vascular disease, peripheral vascular disease), diabetic foot, having hypoglycaemic episodes and uncontrolled glycaemia were not independently associated with LTFU (Table 2).

**Table 2:** Multivariable analysis of predictors of patients lost to follow-up

Variable Name	Category	LTFU N = 1690 n (col%)	uHR 95%CI	P-value	aHR 95%CI	*P*-value
Socio-demographic Characteristics						
Sex						
	Female	712 (42)	*Reference*			
	Male	978 (58)	1.27 (1.15–1.40)	< 0.001	1.17 (1.06–1.30)	**0.002**
Age group						
	19–44	439 (26)	*Reference*			
	45–64	831 (49)	0.73 (0.65–0.83)	< 0.001	0.83 (0.74–0.94)	**0.003**
	Above 64	420 (25)	0.63 (0.55–0.72)	< 0.001	0.78 (0.67–0.90)	**0.001**
Socioeconomic statu						
	Lower	408 (24)	*Reference*			
	Middle	771 (46)	0.88 (0.78–0.99)	0.041	0.83 (0.74–0.94)	**0.003**
	Upper	511 (30)	0.88 (0.77–0.99)	0.046	0.83 (0.73–0.95)	**0.006**
Life Style Characterisitcs						
Smoking						
	No	1509 (89)	Reference			
	Yes	181 (11)	*1.26 (1.08–1.47)*	0.003	1.22 (1.04–1.44)	**0.016**
DM Treatment characteristics						
Lifestyle and monotherapy						
	No	1254 (74)	*Reference*			
	Yes	436 (26)	1.34 (1.20–1.49)	< 0.001	1.02 (0.86–1.22)	0.808
Lifestyle & dual therapy						
	No	740 (44)	*Reference*			
	Yes	950 (56)	0.81 (0.74–0.90)	< 0.001	0.79 (0.67–0.94)	**0.006**
Lifestyle & triple therapy						
	No	1557 (92)	Reference			
	Yes	133 (8)	0.79 (0.66–0.95)	0.01	0.77 (0.61–0.97)	**0.028**
DM Complications and comorbidities						
Hypertension						
	No	593 (35)	*Reference*			
	Yes	1097 (65)	0.71 (0.65–0.79)	< 0.001	0.90 (0.81–1.01)	0.07
Neuropathy						
	No	1141 (68)	Reference			
	Yes	549 (32)	0.64 (0.57–0.70)	< 0.001	0.72 (0.65–0.81)	**< 0.001**
Nephropathy						
	No	1605 (95)	Reference			
	Yes	85 (5)	0.54 (0.43–0.67)	< 0.001	0.57 (0.45–0.71)	**< 0.001**
Retinopathy						
	No	1394 (82)	Reference			
	Yes	296 (18)	0.70 (0.62–0.80)	< 0.001	0.89 (0.78–1.02)	0.101
Macroangiopathy						
	No	1654 (98)	*Reference*			
	Yes	36 (2)	0.75 (0.54–1.05)	0.093	0.91 (0.65–1.27)	0.579
Diabetic foot						
	No	1604 (95)	Reference			
	Yes	86 (5)	0.68 (0.55–0.85)	0.001	0.82 (0.65–1.02)	0.072
Hypoglycaemia						
	No	1651 (98)	*Reference*			
	Yes	39 (2)	0.74 (0.54–1.01)	0.061	0.79 (0.58–1.09)	0.157

Boldface means significant at *P*-value < 0.05 for Tables 1 & 2

## Discussion

In this retrospective study, we found a high proportion of loss to follow up among patients with diabetes mellitus attending a diabetes clinic in a private not for profit urban hospital in Uganda. We found that male gender, having diabetic complications such as neuropathy and nephropathy, and using a triple therapy regimen significantly associated with LTFU. Retention in diabetes care was associated with older age, being in middle and upper socioeconomic status, being a non-smoker and being on a dual therapy regimen. We also found high rates of hypertension and a large number of patients did not achieve the recommended targets for optimal diabetes control.

Our LTFU proportion was much higher than that reported from developed countries which varied from 5.4 to18% in Malaysia, 5.5% in German [13], 12% in Nashville-Tennessee [14] and 46% in Canada [15]. Similarly, our LTFU proportion was higher than that reported from Kenya which had a dropout rate of 31% [22]. Our study was done in a private not for profit hospital where patients pay for their care and that could explain the high proportion of patients lost to follow up. Although the LTFU might be due to deaths being misclassified as LTFU the cost of medical care could have resulted in the majority of patients with diabetes mellitus self-refering themselves to other public facilicties where care is free, without proper referral procedures. This is supported by the findings that those with middle and higher socioeconomic status and thus more able to meet the medical care costs were more likely to be retained compared to the lower socioecomic status. However since deaths and transfer outs were not recorded, the resultant misclassification might have led to a biased high estimate of the LTFU as high mortality rates among diabetes patients have previously been reported from some studies [27–29].

In our study, we found that male gender, was associated with loss to follow up, similar findings were reported by Chew et al. who analysed  the diabetes mellitus registry in Malaysia [12]. Other chronic care treatment providers like HIV programs have also reported that males were associated with clinic non-attendance. Additionally, being on a triple therapy regimen and having had complications of diabetes mellitus like neuropathies and nephropathy were also associated with loss to follow up. However, if the patients with diabetes complications and those on triple therapy could have died, this could have  possibly lead to a biased overestimate of LTFU. These findings are similar to those that were  reported by Chew et al., in Malaysia and similar to other quantitative studies done in the United Kingdom and United States that showed presence of neuropathy, low income, smoking and male sex being associated to loss to follow up.

### Study strengths

To our understanding, this study is one of the first studies to evaluate diabetes care in a private not for profit health facility in Uganda focusing on patients’ LTFU. Assessment of LTFU among patients with diabetes mellitus is important as it is directly related to attainment of optimal glyceamic control and prevention of complications of diabetes. Secondly understanding factors associated with LTFU from care can provide valuable information for the improvement of care. The use of patients routine care data for this review has provided us with a large sample size which would not have been feasible if otherwise.

### Study limitations

As with most clinic care records, the quality of data recorded is usually less satisfactory, and in our case, the incomplete data on referrals and deaths might have biased our estimated LTFU. Additionally this  data from an operational program was initially not designed for research purposes, not validated, had no quality checks done for completeness and plausibility, and we were only able to look at a limited number of variables documented in the patient’s charts. We also have little understanding of the health system and patient related reasons for drop out of care which can only be answered by a purposively designed qualitative study. Due to the changes in the general economic status in the country, the patients economic status might have changed over the 13 years duration of the study from what it was at registration in the clinic. Although rural to urban migration is common among those searching for jobs, urban to rural migration also occurs among those who find urban employment and survival hard to obtain, which might have contributed to LTFU. The method used by the health facility to classify socioeconomic status and residence were not standardised and could have changed over the years.

## Conclusion

Loss to follow up in a diabetes mellitus care has serious implications for patients with diabetes mellitus due to the subsequent complications and resultant morbidity, mortality and management costs as well as the disease burden on the health system. Our findings warrant stengthening patient follow up in diabetes care and conducting interventional studies targeting the identified associated risk factors for LTFU.

## Data Availability

Data will not be shared publicly due to the data sharing policy of the MRC/UVRI and London School of Hygiene and Tropical Medicine Uganda Research Unit, which requires a prior data sharing agreement. However, a full data set containing the data supporting the study findings in this report can be obtained from the Unit Director, by email to: mrc@mrcuganda.org or the corresponding author. More clarification on this can be accessed through this website: https://www.mrcuganda.org/publications/data-sharing-policy

## Abbreviations

aHR: Adjusted Hazard Ratio
CI: Confidence intervals
H3A: Human Heredity and Health in Africa
HbA1c: Glycated haemoglobin
HRs: Hazard Ratios
Km: Kilometer
LDL-C: Low Density Lipoprotein cholesterol
LRT: Likelihood ratio tests
LTFU: Loss to follow up
REC: Research and Ethics Committee
SES: Socioeconomic Status
uHR: Unadjusted hazard ratio
UNCST: Uganda National Council for Science and Technology
UVRI: Uganda Virus Research Institute
